# The Clinical and Vascular Characteristics of RNF213 c.14576G>A Variant-Related Intracranial Major Artery Disease in China

**DOI:** 10.1155/2019/7908392

**Published:** 2019-03-12

**Authors:** Weiyang Cheng, Sufang Xue, Fang Wu, Xiaowei Song, Qiang Huang, Haiqing Song, Jian Wu

**Affiliations:** ^1^Beijing Tsinghua Changgung Hospital, School of Clinical Medicine, Tsinghua University, Beijing, China; ^2^Dongguan People's Hospital (Affiliated Dongguan Hospital, South Medical University), Dongguan, Guangdong Province, China; ^3^Department of Neurology, Xuanwu Hospital of Capital Medical University, Beijing, China; ^4^Department of Radiology, Xuanwu Hospital of Capital Medical University, Beijing, China; ^5^Center of Stroke, Beijing Institute for Brain Disorders, China

## Abstract

**Background and Purpose:**

Recently, several studies indicated the c.14576G>A variant on the ring finger protein 213 (RNF213), a founder variant of moyamoya diseases (MMD), was associated with non-MMD intracranial major artery stenosis/occlusion (non-MMD ICASO). We proposed that RNF213 variant-related ICASO including MMD might be a special entity with its own characteristics based on a genetic background. The aim of the study was to learn the clinical and vascular features of RNF213 variant-related ICASO. Moreover, we tried to explore the clinical significance of a testing variant in ICASO patients in China.

**Methods:**

Clinical material and routine image data were collected in 160 Chinese patients with ICASO, including 41 verified MMD and 119 non-MMD. DNA samples were extracted, and the c.14576G>A variant on RNF213 was genotyped. Then, the clinical and vascular features were compared between the patients with and without a relevant variant. Furthermore, the patients with RNF213 mutation were performed with high resolution magnetic resonance imaging (HR-MRI) examination to conclude features of the artery wall.

**Results:**

There were 16 (10%) patients (including 9 MMD and 7 non-MMD ICASO) presenting a heterozygous c.14576G>A variant while none of homozygote was found. Compared to the patients without the c.14576G>A variant, the variant group had more female, less symptomatic patients, and more possibility of having collateral vessels in vascular imaging. In the symptomatic subgroup, there is no significant difference in clinical presentation (*p* > 0.05) between two groups. However, RNF213 variant-related ICASO had lower scores in NIHSS (1.0 ± 3.0 vs. 3.9 ± 5.0, *p* < 0.05) but not in mRS. In the symptomatic subgroup, in addition, most of the HR-MRI images of variant ICASO (77.8%, 7 of 9) were characterized by a shrunken outer diameter, concentric thickening vessel wall, and collateral vessel structures on the stenotic portion, which was prone to be diagnosed as HR-MMD (a MMD diagnosis diagnosed by HR-MRI). The rest of the two variants showed a relatively eccentric luminal narrow, normal outer diameter without collateral vessel findings, identified as HR-ICAD (intracranial atherosclerotic disease diagnosed by HR-MRI).

**Conclusions:**

Our study demonstrated that the c.14576G>A variant on RNF213 may be a biomarker to good outcome of ICASO in Chinese. The variant-related ICASO was characterized by both features of MMD and ICAD diagnosed by HR-MRI.

## 1. Introduction

Intracranial major artery stenosis/occlusion (ICASO) is a common cause of stroke worldwide [1], especially in Asian, Black, and Hispanic ancestries [2–4]. There are some etiologies which can lead to ICASO, such as atherosclerosis, moyamoya disease (MMD), dissection, and vasculitis [5]. Histopathologically, atherosclerotic intracranial disease (ICAD) is different from MMD, which was characterized by progressive stenosis or occlusion of the distal internal carotid artery (ICA), proximal MCA, and/or ACA with the development of a thin collateral network of small vessels at the base of the brain [6]. Although ICAD and MMD have different pathologies, it was hard to identify from each other by clinical and routine vessel examination.

Ring finger protein 213 (RNF213), especially the mutation of c.14576G>A, was proven to be strongly associated with MMD in Asian population, including Japanese, Korean, and Chinese [6]. Furthermore, Miyawaki et al. indicated ICASO without signs of MMD (non-MMD ICASO) was also related to the c.14576G>A variant in RNF213 [7, 8]. Subsequently, Bang et al. not only repeatedly certified this view in Korean population [9] but also found that some RNF213 variant-related non-MMD ICASO could develop to MMD during follow-up [10]. This association of the c.14576G>A variant in RNF213 with non-MMD ICASO also has been proved in Chinese population by our previous study (unpublished data). Some researchers supposed that non-MMD with the c.14576G>A variant may be the MMD in an early stage so that we can differentiate early MMD from ICAD only by using genetic analysis [10]. However, Bang et al. recently discovered RNF213 is also a susceptibility gene for ICAD, which was confirmed by conventional angiography (absence of basal collaterals) and high-resolution MRI (HR-MRI, presence of plaque) in East Asians [11]. Therefore, only screening the RNF213 c.14576G>A variant could not accurately differentiate early MMD from atherosclerotic ICASO.

In this study, we supposed that the RNF213 c.14576G>A variant-related ICASO, including MMD, might be a special spectrum of ICASO, which has their own clinical and vascular characteristics based on the genetic background. Thus, we compared the clinical and vascular features between the ICASO patients with and without a relative variant. HR-MRI examination was subsequently performed in the mutational group to explore the clinical significance by screening the c.14576G>A variant in ICASO.

## 2. Methods

### 2.1. Subjects

Subjects were the recruited patients who visited our hospital from October 2012 to February 2016. The inclusion criteria were as follows: (1) all participants were diagnosed with significant (≥50%) stenosis or occlusion on the distal of the internal carotid artery (ICA) and/or the middle cerebral artery (MCA) and/or the anterior cerebral artery (ACA), with/without a collateral network near the stenosis/occlusion. (2) They were ≤60 years old. (3) They have underwent two or more checkups by transcranial Doppler (TCD), magnetic resonance angiography (MRA), computed tomography angiography (CTA), or digital subtraction angiography (DSA). Subjects were diagnosed as MMD when they satisfied the guidelines of the Research Committee on Spontaneous Occlusion of the circle of Willis [12], and the rest of them were diagnosed as non-MMD ICASO. Patients were excluded when (1) they were found with extracranial or vertebrobasilar stenosis. (2) They were identified with cardiac embolism, dissection, or other stroke mechanisms. The local ethics committee approved our study, and all participants were provided written informed consent.

### 2.2. Collection of Clinical and Radiographic Imaging Data

Clinical materials were collected from medical charts including inpatients and outpatients. Clinical information included demographic material and vascular risk factors [including hypertension, diabetes, hyperlipidemia, cardiopathy, family history of cerebrovascular disease (CVD), smoking, and drinking]. According to the clinical presentation, we subgroup the symptomatic patients into three types, namely, ischemia, hemorrhage, and mixture. In addition, NIHSS scores (the latest stroke episode) and mRS scores (three months after the latest stroke episode) were collected. Hypertension, diabetes, and hyperlipidemia were defined according to the relevant guidelines [13–15]. The images of MRA/CTA/DSA were analyzed by at least one radiologist and at least one neurologist. Vessel involvement [including the terminal internal carotid artery (TICA), anterior cerebral artery (ACA), middle cerebral artery (MCA), and posterior cerebral artery (PCA), unilateral or not] and the existence of collateral vessels (moyamoya vessels identified by MRA or DSA) were collected and evaluated.

### 2.3. Mutation Screening

DNA samples were extracted from peripheral blood lymphocytes by standard phenol/chloroform extraction methods. Primers were designed to amplify the target region spanning c.14576G>A to identify RNF213 polymorphisms. Primers were as follows: forward 5′ GCTGGTAAAGTTCCTGCCTG 3′ and reverse 5′ CTGTTCCCCTATGCA GTGATC 3′. The PCR was performed under the condition as follows: denaturation at 95°C for 5 min, then 30 cycles of 94°C for 15 seconds, 56°C for 15 seconds, 72°C for 15 seconds, and final extension at 72°C for 10 minutes. The PCR products were purified using a commercial kit (OMEGA D2500-01) and were directly sequenced at BGI in Beijing.

### 2.4. Imaging Protocol and Analysis of HR-MRI

We have described the imaging protocol of HR-MR before (unpublished data). In brief, subjects enrolled were scanned with a 3.0-TMR imager (Magnetom Verio; Siemens, Erlangen, Germany). Standard MR imaging protocol underwent included precontrast and postcontrast 3D sampling perfection with application-optimized contrasts by using different flip angle evolutions (SPACE) and 3D time-of-flight (TOF) MR angiographic sequences. The 3D TOF MR imaging was acquired by using the following parameters: repetition time msec/echo time msec, 20/3.6; field of view, 220 × 220 mm; slice thickness, 0.7 mm; and voxel size, 0.7 × 0.7 × 0.7 mm. The 3D SPACE MR imaging was acquired by using the following parameters: repetition time msec/echo time msec, 900/15; field of view, 170 × 170 mm; slice thickness, 0.53 mm; and voxel size, 0.5 × 0.5 × 0.5 mm.

All of the HR-MRI images were analyzed by consensus of a neurologist and a radiologist who were blinded to the clinical materials of the patients. Two reviewers evaluated the characteristics including the outer diameter, wall thickening pattern, signal intensity, enhancement, pattern of enhancement, and collateral vascular structures near the stenotic part. These characteristics were evaluated using the parameters previously described [13–15], and the parameters are as follows: (1) outer diameter—the remodeling index (RI) refers to the ratio of the vessel area at the MCA to the contralateral MCA or midbasilar artery when the bilateral MCA was abnormal, and when RI < 0.9, the MCA was thought to have a shrunken outer diameter. (2) Wall thickening pattern—when the vessel wall of the most stenotic part thickens circumferentially, we defined it as concentricity; otherwise, defined as eccentricity. (3) The presence and pattern of enhancement—enhancement was estimated by comparing the precontrast and postcontrast sagittal SPACE images. Concentric enhancement was defined as circumferential contrast. Eccentric enhancement was regarded as contrast without 360° circumferential or when the thickest part was more than twice the thinnest part where circumferential enhancement existed. (4) Collateral vascular structures—it was defined as two or more small round vascular structures near the stenotic part in the HR-MRI image. Based on the above parameters, presumptive diagnosis was made similar to the previous study [10]: namely, (1) an intracranial atherosclerotic disease diagnosed by HR-MRI (HR-ICAD) shows an eccentric plaque, without a shrunken outer diameter and collateral vascular structures. (2) A MMD diagnosis diagnosed by HR-MRI (HR-MMD) refers to the concentric shrinkage of the vessel wall, shrunken outer diameter, and collateral vascular structures, sometimes accompanying diffuse concentric enhancement on the affected segment.

### 2.5. Statistical Analysis

All the analyses were performed by IBM SPSS 19.0 software. Quantitative data was expressed as the mean ± standard deviation ($\bar{x}\pm$). Qualitative data was summarized using counts and percentages. Statistical calculations for quantitative variables were compared using a *t*-test or continuous Kruskal-Wallis test (nonnormally distributed), and a chi-square test or Fisher's exact test was used to analyze the qualitative data. Bilateral *p* value < 0.05 was considered statistically significant.

## 3. Results

### 3.1. Clinical Characteristics Related with RNF213 Variant-Related ICASO

A total of 160 ICASO patients were enrolled in our study. 16 (10%) of the participants presented a heterozygous c.14576G>A variant while none of homozygote was found.

Then, clinical characteristics were compared between patients with a RNF213 c.14576G>A variant (*n* = 16) and without (*n* = 144). The rate of female was significantly higher (56.3% vs. 24.3%, *p* < 0.05), and there were less symptomatic patients (56.3% vs. 88.9%, *p* < 0.05) in the variant group (as shown in Table 1).

There were 9 and 128 symptomatic patients in the group with the RNF213 variant and without, respectively. In this symptomatic subgroup, there was no significant difference in clinical presentation (*p* > 0.05) between two groups. The group with the variant possessed a less NIHSS score, but not a mRS score (NIHSS: 1.0 ± 3.0 vs. 3.9 ± 5.0, *p* < 0.05; mRS: 1.0 ± 0.87 vs. 1.5 ± 1.46, *p* > 0.05) (as shown in Table 2).

### 3.2. Vascular Feature in RNF213 Variant-Related ICASO

**Figure 1:**
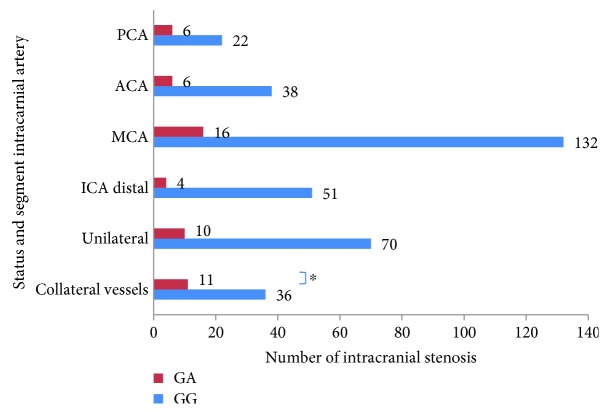
The number of intracranial stenotic distribution and status in two groups. ^∗^*p* value < 0.05.

Compared with the nonvariant group, the collateral vessels were more common in the RNF213 mutational group (68.6% vs. 25%, *p* < 0.05). However, no significant difference was found in the intracranial artery involvement and unilateral involvement (as shown in Table 3, Figure 1).

### 3.3. Vessel Wall Features of HR-MRI in RNF213 Variant-Related ICASO

**Figure 2:**
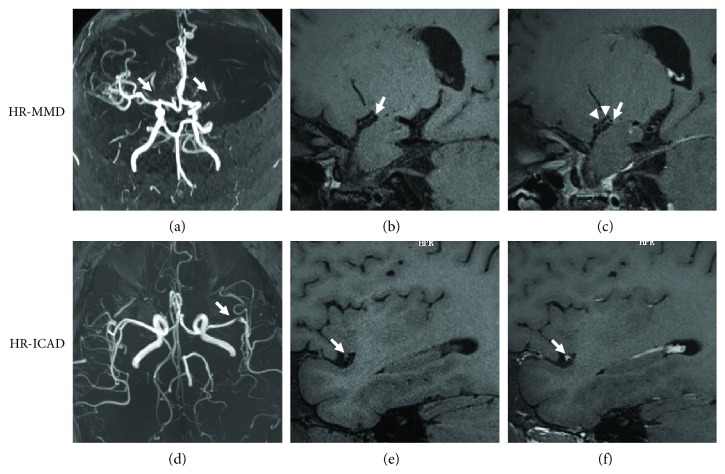
Images of HR-MMD and HR-ICAD patients with RNF213 c.14576G>A variant. (a–c) A 44-year-old woman with asymptomatic MMD, diagnosed as HR-MMD. TOF MRA showed the occlusion of the left MCA and moderate to severe stenosis of right MCA (a). A precontrast image of the right MCA reveals a small outer diameter with concentric wall thickening (b) and concentric enhancement on the vessel wall in a postcontrast image with collateral vessels (c). (d–f) A 36-year-old man with ICAD presented with cerebral infarction, diagnosed as HR-MMD. TOF MRA demonstrated severe stenosis of the left MCA (d). A precontrast image of the left MCA showed eccentric stenosis and a normal outer diameter (e) with eccentric plaque enhancement in a postcontrast image (f).

9 of 16 patients with RNF213 variant-related ICASO underwent HR-MRI examination (4 of the rest refused this examination; 2 of the rest could not complete because of severe illness; and 1 of the rest cannot be contacted). Most of them (77.8%, 7/9) are characterized by a shrunken outer diameter, concentric thickening vessel wall, and collateral vessel structures on the stenotic part, supposed to be MMD based on HR-MRI (HR-MMD). After contrast injection, 4 of them showed concentric homogeneous enhancement and one patient did not show enhancement. Contrarily, the rest 2 patients showed relatively eccentric luminal narrowing, a normal outer diameter without collateral vessel findings, identified as ICAD based on HR-MRI (HR-ICAD). Both of them revealed eccentric enhancement on the segment of stenosis/occlusion (as shown in Table 4, Figure 2).

## 4. Discussion

Our study took initiative to describe the features of RNF213 c.14576G>A variant-related ICASO. The major findings are as follows: (a) the ICASO with the RNF213 c.14576G>A variant possessed less symptomatic patients and milder presentation compared with wild-type genotype; (b) collateral vessels were more common in ICASO with RNF213 variant; (c) ICASO with the RNF213 variant was characterized by features of both HR-MMD and HR-ICAD.

RNF213 encoded a protein with 5256 amino acid, which functioned as E3 ubiquitin ligase activity and energy-dependent unfoldase [16]. The mutation of c.14576G>A in RNF213 was proven to be strongly associated with MMD in Asian population [16–18]. Furthermore, Miyawaki et al. and Bang et al., respectively, revealed the closely relationship between the c.14576G>A variant in RNF213 and non-MMD ICASO in Japanese and Korean populations. Our previous study also proved this view in Chinese population (unpublished data). Non-MMD ICASO with RNF213 mutation was identified as MMD in the early stage, but surprisingly, Bang et al. have recently discovered RNF213 is also a susceptibility gene for ICAD, confirmed by conventional angiography and HR-MRI in East Asians [11]. Therefore, parts of MMD and ICAD have a common genetic background, which was supposed to be a new spectrum named the RNF213 c.14576G>A variant-related ICASO.

Miyatake et al. confirmed that MMD with homozygous c.14576G>A in RNF213 was significantly associated with an earlier age at onsets and more infarctions as the first symptom [19]. Therefore, they proposed the homozygous c.14576G>A genotype to be a biomarker predicting the severe type of MMD and finding patients who should require early operation in Japanese population [19]. Subsequently, the same conclusion was taken in Korean patients with MMD [20]. Chinese researchers Wu et al. have found that RNF213 mutation of c.14576G>A, mainly a heterogeneous variant, was particularly related to ischemic type MMD, whereas mutation of A4399T was associated with hemorrhage in Han Chinese population [21]. All of these studies referred to phenotype-genotype correlation in MMD. Recently, Bang et al. found that RNF213 variant carriers of ICAD were younger and had higher rates of family history than noncarriers. This was the first report that shown the correlation between RNF213 genotype and phenotype in ICAD [11].

Our study demonstrated that RNF213 c.14576G>A variant-related ICASO possessed less symptomatic patients and milder presentation compared with wild-type genotype. In other words, our study showed that mutation in RNF213 could be a biomarker to asymptomatic or mild outcome of ICASO patients. However, our conclusion was contrary to those of the previous studies [19, 20]. There were several reasons for the difference. Firstly, a nonhomogeneous genotype was detected in our subject while previous finding was referred to the homogeneous genotype. We need more studies to reveal the relationship between the homozygous c.14576G>A variant and phenotype of ICASO in Chinese. Secondly, it can be attributed to the different standard of enrollment. Both MMD and ICAD were enrolled in the mutation group in our study, which was different to pure MMD in previous studies. Lastly, the genotype and phenotype relationship varied from diverse races. We supposed that the less proportion of symptomatic patients and milder presentation may be related to the compensation of collateral vessels, which were more common in the RNF213 c.14576G>A variant in our study. Previously, Liu et al. [16] indicated that RNF213 c.14576G>A variant-related zebrafish shaped irregular wall formation and abnormal sprouting vessels. However, in our study, there was no difference in the symptomatic subgroup in presentation, which was different from Wu et al.'s study in Chinese population [21]. The difference may be attributed to our small samples and different participants. More large multicentral studies are needed. HR-MRI was known to be an advance imaging technique to observe vessel wall changes and to identify MMD from ICAD [10, 22, 23]. In our study, 9 mutational patients underwent imaging by 3T HR-MRI to investigate the feature of the vessel wall. Our study found that part of this new entity, c.14576G>A variant-related ICASO, was characterized by concentric shrinkage, smaller outer diameter with well-developed collateral vessels, regarded as HR-MMD. Part of variant RNF213 showed relatively eccentric luminal narrowing, normal outer diameter with eccentric enhancement, considered to be HR-ICAD. Therefore, the c.14576G>A variant in RNF213 was associated with not only MMD but also ICAD, which was compatible with the previous study [11]. It is reasonable to view the c.14576G>A variant-related ICASO as an entity, including ICAD and MMD. Among 5 HR-MMD patients which obtained enhanced images, 4 of them showed concentric homogeneous enhancement and one patient did not show enhancement. Interestingly, only one patient without enhancement did not have any atherosclerotic risk factors, while enhanced ones had at least one or more risk factors. The mechanism of enhancement of the vessel wall was not clearly understood, but it was more frequently detected in the vessel wall responsible for stroke [24]. We supposed that inflammatory components caused by vascular risk factors may be related to the enhancement of the vascular wall. Histopathology must be elucidated in future researches.

There are limitations in this study. Firstly, the sample size in our study is too small, especially the ICASO patients with the RNF213 variant. More large trials are needed. Secondly, HR-MRI could not describe the true histopathology of the vessel wall, which does not have acknowledged diagnostic standard for ICAD and MMD. Lastly, our study is just a pilot study so it could not be a representative of the general population. The conclusions should be certified by multicenter randomize controlled trials afterwards.

## 5. Conclusion

Our study demonstrated that the RNF213 c.14576G>A variant may be a biomarker to the good outcome of ICASO in Chinese population. RNF213 c.14576G>A variant-related ICASO was characterized by both features of MMD and ICAD diagnosed by HR-MRI.

## Figures and Tables

**Table 1:** Clinical characteristic comparison of patients with different genotypes.

Characteristics	Genotype		*p* value	*Z*/*χ*^2^
	Nonvariant (144)	Variant (16)		
Female gender (%)	35 (24.3)	9 (56.3)	0.016^∗^	5.855
Age at diagnosis	44 ± 9.97	46.1 ± 8.24	0.504	-0.669
Symptomatically (%)	128 (88.9)	9 (56.3)	0.002^∗^	9.952
Hypertension (%)	54 (37.5)	9 (56.3)	0.145	2.121
Diabetes (%)	22 (15.3)	3 (18.8)	1.000	<0.001
Hyperlipidemia (%)	51 (35.4)	7 (43.8)	0.511	0.433
Cardiopathy (%)	5 (3.5)	1 (6.3)	1.000	<0.001
Family history of CVD (%)	46 (31.9)	4 (25.0)	0.570	0.323
Smoking (%)	71 (49.3)	4 (25.0)	0.065	3.416
Drinking (%)	50 (34.7)	4 (25.0)	0.435	0.609

Note: ^∗^*p* value < 0.05.

**Table 2:** Comparison of clinical presentation of patients between different genotypes in the symptomatic subgroup.

Characteristics		Genotype		*p* value	*Z*/*χ*^2^
		Nonvariant (128)	Variant (9)		
Symptomatic types	Ischemia	124 (96.9)	8 (88.9)	0.328	2.227
	Hemorrhage	2 (1.6)	1 (11.1)		
	Mixture	2 (1.6)	0 (0)		
NIHSS score		3.9 (5.0)	1.0 (3.0)	0.008^∗^	-20672
mRS score		1.5 (1.46)	1.0 (0.87)	0.057	-0.938

Note: ^∗^*p* value < 0.05.

**Table 3:** Intracranial artery stenotic features of two genotypes.

CTA/MRA/DSA	Genotype		*p* value	*Z*/*χ*^2^
	Nonvariant (144)	Variant (16)		
TICA	51 (35.4)	4 (25)	0.405	0.693
MCA	132 (91.7)	16 (100)	0.484	0.490
ACA	38 (26.4)	6 (37.5)	0.516	0.421
PCA	22 (15.3)	6 (37.5)	0.061	4.291
Unilateral	70 (48.6)	10 (62.5)	0.292	1.111
Collateral vessels	36 (25)	11 (68.6)	0.001^∗^	11.260

Note: ^∗^*p* value < 0.05.

**(a):** 

Sample	Genotype	Sex	Age at onset	Nation	Risk factor	Blood tests	Symptomatic	First presentation	Type of first presentation	Presumed diagnosis by MRA/DSA
1	GA	F	44	Han	HT, DM, FH	Normal	N	——	——	MMD
2	GA	M	53	Han	HT, FH, smoking, drinking	Normal	Y	Headache, visual field defects, dizziness	Cerebral infarction	MMD
3	GA	M	54	Han	HT, FH, smoking, drinking	HHcy	N	——	——	ICAD
4	GA	F	45	Han	HT	Normal	N	——	——	ICAD
5	GA	F	58	Han	HT, HL	Normal	N	——	——	ICAD
6	GA	F	51	Han	HL	Normal	N	——	——	ICAD
7	GA	F	54	Han	HT	Normal	Y	Paroxysmal dysphonia and weakness of the right extremities	TIA	ICAD
8	GA	M	36	Han	HT, HL, smoking	Normal	Y	Sudden dizziness, dysphonia and blunt reaction	Cerebral infarction	ICAD
9	GA	M	50	Han	NO	Normal	N	——	——	ICAD

**(b):** 

Sample	HR-MRI							
	TOF	Outer diameter	Wall thickening pattern	Enhancement on vessels	Pattern of enhancement	Signal intensity	Collateral vascular structures	Diagnosis by HR-MRI
1	Bilateral ACA and MCA disappeared, replaced by moyamoya vessels	Shrinkage	Concentric	Y	Concentric	Homogeneous	Y	HR-MMD
2	Bilateral ACA and MCA disappeared, replaced by moyamoya vessels	Shrinkage	Concentric	Y	Concentric (eccentric enhancement on the MCA distal)	Homogeneous	Y	HR-MMD
3	Moderate stenosis in M2 of left MCA	Normal	Eccentric	Y	Eccentric	Homogeneous	N	HR-ICAD
4	Occlusion of left MCA, moderate to severe stenosis of right MCA	Shrinkage	Concentric	Y	Concentric	Homogeneous	Y	HR-MMD
5	Mild stenosis of left MCA, occlusion of right MCA	Shrinkage	Concentric	—	—	Homogeneous	Y	HR-MMD
6	Moderate stenosis of right MCA	Shrinkage	Concentric	Y	Concentric	Homogeneous	Y	HR-MMD
7	Occlusion of left MCA, mild stenosis of right MCA	Shrinkage	Concentric	—	—	Homogeneous	Y	HR-MMD
8	Severe stenosis of left MCA	Normal	Eccentric	Y	Eccentric	Homogeneous	N	HR-ICAD
9	Occlusion of left MCA	Shrinkage	Concentric	N	Concentric	Homogeneous	Y	HR-MMD

Note: F: female; M: male; HT: hypertension; HL: hyperlipidemia; DM: diabetes mellitus; FH: family history; HHcy: hyperhomocysteinemia; Y: yes; N: no.

## Data Availability

All data generated or analyzed during this study are included within the article.
